# The role of GRIP1 and ephrin B3 in blood pressure control and vascular smooth muscle cell contractility

**DOI:** 10.1038/srep38976

**Published:** 2016-12-12

**Authors:** Yujia Wang, Zenghui Wu, Hongyu Luo, Junzheng Peng, John Raelson, Georg B. Ehret, Patricia B. Munroe, Ekatherina Stoyanova, Zhao Qin, Guy Cloutier, W. Edward Bradley, Tao Wu, Jian-Zhong Shen, Shenjiang Hu, Jiangping Wu

**Affiliations:** 1 Research Centre, Centre hospitalier de l’Université de Montréal (CHUM), Montreal, Quebec H2X 0A9, Canada; 2 Center for Complex Disease Genomics, McKusick-Nathans Institute of Genetic Medicine, Johns Hopkins University School of Medicine, Baltimore, Maryland 21205, USA; 3 Clinical Pharmacology and The Genome Centre, William Harvey Research Institute, Barts and The London School of Medicine and Dentistry, Queen Mary University of London, London EC1M 6BQ, UK; 4 Institute of Cardiology, First Affiliated Hospital, Zhejiang University Medical College, Hangzhou, 310003, China; 5 Nephrology Service, Centre hospitalier de l’Université de Montréal (CHUM), Montreal, Quebec H2X 0A9, Canada

## Abstract

Several erythropoietin-producing hepatocellular receptor B family (EPHB) and their ligands, ephrinBs (EFNBs), are involved in blood pressure regulation in animal models. We selected 528 single nucleotide polymorphisms (SNPs) within the genes of *EPHB6, EFNB2, EFNB3* and *GRIP1* in the EPH/EFN signalling system to query the International Blood Pressure Consortium dataset. A SNP within the glutamate receptor interacting protein 1 (*GRIP1)* gene presented a *p*-value of 0.000389, approaching the critical *p*-value of 0.000302, for association with diastolic blood pressure of 60,396 individuals. According to echocardiography, we found that *Efnb3* gene knockout mice showed enhanced constriction in the carotid arteries*. In vitro* studies revealed that in mouse vascular smooth muscle cells, siRNA knockdown of GRIP1, which is in the EFNB3 reverse signalling pathway, resulted in increased contractility of these cells. These data suggest that molecules in the EPHB/EFNB signalling pathways, specifically EFNB3 and GRIP1, are involved blood pressure regulation.

Erythropoietin-producing hepatocellular receptor (EPH) kinases are the largest family of receptor tyrosine kinases. They are divided into A and B subfamilies according to sequence homology^1^. Ephrins (EFNs), which are also cell surface molecules, are ligands of EPHs. EFNs are classified as A and B subfamilies. EFNAs attach to the cell surface through glycosylphosphatidylinositol anchoring, whereas EFNBs attach through transmembrane sequences^2^,^3^,^4^. Interactions among EPHs and EFNs are promiscuous but, in general, EPHA members interface preferentially with EFNAs, and EPHBs with EFNBs^2^,^3^,^4^. Such redundancy suggests that these kinases are crucial in various biological contexts. EFNs can stimulate EPH receptors, and this is called forward signalling. Interestingly, EPHs are also capable of stimulating EFNs which then transmit signalling reversely into cells, a phenomenon known as reverse signalling.

EPHs and EFNs are expressed in many tissues and organs. They play important roles in the central nervous system^2^,^4^, immune system^5^,^6^,^7^,^8^,^9^,^10^,^11^,^12^,^13^,^14^, digestive system^15^, bone metabolism^16^,^17^, angiogenesis^18^ and other processes^19^,^20^,^21^.

We recently reported that EPHB6, in concert with sex hormones, is crucial in VSMC contraction and blood pressure (BP) regulation^22^. *Ephb6* gene knockout (KO) mice after castration manifest higher blood pressure that their wild type (WT) counterparts^22^. Vascular smooth muscle cells (VSMC) are a target tissue through which EPHB6 exerts its effect on BP control. Since EPHB6 and all its major ligands of the EFNB family, *i.e.,* EFNB1, EFNB2 and EFNB3, are expressed in VSMCs^22^, there is a molecular framework for their function in these cells.

We showed that while solid-phase recombinant EPHB6 reduces VSMC contraction in response to phenylephrine (PE) stimulation, solid-phase anti-EPHB6 antibody (Ab) does not^22^, indicating that reverse signalling from EPHB6 to EFNBs but not forward signalling from EFNBs to EPHB6 is responsible for dampening VSMC contractility. Since all the cells in the vascular bed express EPHB6 and the 3 EFNB ligands, EPHB6 expressed in neighboring VSMCs can trigger reverse signaling of a certain EFNBs in a VSMC. For EPHB6, such reverse signaling has a default function of reducing VSMC contractility, leading to lower BP. In the absence of such reverse signaling, such as the case of EPHB6 KO, the VSMC contractility will increase, resulting higher BP.

In support of this notion, we have shown that deletion of EFNB1, a ligand of EPHB6, results in a hypertensive phenotype in mice^23^. Therefore, we identified EPHB6 and, by logical extension and with certain experimental evidence, its ligands (EFNBs) as novel BP regulatory factors in animal models.

In order to establish the relevance of our findings in mice to human blood pressure regulation, we selected 528 single nucleotide polymorphisms (SNPs) within the genes of *EPHB6, EFNB2, EFNB3* and glutamate receptor interacting protein 1 (*GRIP1)*, which is a key molecule in EFNB reverse signalling, to query the International Blood Pressure Consortium (IBPC) dataset, which contains SNP information on 69,395 individuals. We found that a SNP in the *GRIP1* gene approached statistical significance for association with diastolic blood pressure in humans. Additional animal studies revealed roles of EFNB3 and GRIP1in regulating arterial tone and VSMC contractility, providing phenotypic evidence supporting the genetic findings in humans.

## Materials and Methods

### Meta-analysis of SNPs in EPHB and EFNB genes and a related gene GRIP1 for association with BP phenotypes in humans

The *p*-values for association with diastolic pressure (DP) and systolic pressure (SP) were calculated for a total of 528 SNPs found within the regions of 4 genes (*EPHB6, EFNB2, EFNB3* and *GRIP1)* and within 10 kb 5′ and 3′ of these genes, employing the LocusZoom genome browser^24^ to query the IBPC dataset^25^, which contains SNP information on 69,395 individuals of European ancestry in 29 general population-based cohorts.

These 528 SNPs represented 166 independent linkage disequilibrium (LD) blocks, as determined by the Tagger program^26^ on the HapMap website^27^. Table 1 lists the genes and regions in which the SNPs are located. Query of 166 independent LD blocks resulted in a Bonferroni-corrected critical *p*-value of 0.0003012 for a given BP phenotype (systolic or diastolic pressure). Looking for the best results between systolic or diastolic pressure phenotypes would require a lower critical *p*-value; however, correcting for 2 × 166 tests (p = 0.000151) would be overly conservative as these 2 measurements are not independent.

This human genetic study was carried out in accordance with relevant guide lines of the participating institutions. The research protocol was approved by the institutional ethics committees of the institutions. Informed consent was obtained from all the subjects used in this study.

### Efnb3 KO mice

*Efnb3* KO mice were produced by Regeneron Pharmaceuticals, as described previously^28^, and generously provided to us. They had been backcrossed to the C57BL/6 background for more than 10 generations. Age- and gender-matched WT littermate mice served as controls and are referred to as WT mice. The mice were housed in ambient temperature (22 °C) with 12-h light and dark cycles. For all the *in vivo* experiments, mice were housed one per cage.

### Echocardiography

Transthoracic echocardiography was undertaken in mice lightly anesthetized with isoflurane. The experiments were always conduced around noon time. The oestrus cycles of the females were not monitored. Their carotid vessels and heart were imaged with a high-resolution ultrasound biomicroscope (Vevo770; Visualsonics, Toronto, ON, Canada) equipped with a 100% bandwidth 30-MHz central frequency transducer (RMV-707, 12.7 mm focal length, 6 mm aperture). Lateral and axial resolutions with this probe are ~115 μm and ~55 μm, respectively^29^. Preheated ultrasound transmission gel (Aquasonic 100, Parker Laboratories, Orange, NJ, USA) was placed on regions of interest to provide acoustic coupling medium between the transducers and animals. The left and right common carotid arteries were imaged longitudinally in B mode to guide recordings of Doppler time-varying flow velocities for 2 s. Doppler sample volume was positioned 1–2 mm prior to the carotid bifurcation at a 60° angle. Acquired angle-corrected Doppler data were analyzed to measure the mean Pourcelot index (PI) over 10 consecutive cardiac cycles, according to Stoyanova *et al*.^30^. The PI is a dimensionless echocardiographic parameter that characterizes vascular hemodynamics downstream of a measurement point. It depends on both arterial compliance and downstream vascular resistance.

The heart was also imaged in B mode via the parasternal long-axis view to assess aortic hemodynamics and cardiac output (CO). The M-mode sampling line was positioned perpendicularly to the ascending aorta, 0.5–1.5 mm downstream of the aortic valve, and time-varying tracings tracked changes in aortic diameter (AoD). Mean AoD was assessed over 5 consecutive cardiac cycles. Doppler velocity waveforms were then recorded in the ascending aorta by positioning sampling volume at the exact same location where M-mode tracings were obtained. The envelope of angle-corrected (60°) Doppler tracings was delineated manually to compute the velocity time integral (VTI), which was averaged over 10 cardiac cycles. Assuming a parabolic velocity profile in the ascending aorta^30^, stroke volume (SV) was calculated (in ml) as ½ (AoD/2)^2^ × π × VTI, and CO (in ml/min) was estimated as SV × heart rate (HR), where HR was mean HR of the animals.

Left ventricle (LV) dimensions (in mm) at end-systole and end-diastole were finally assessed to quantify fractional shortening, ejection fraction and LV mass. B-mode parasternal long-axis viewing guided the capture of M-mode tracings through the anterior and posterior LV walls at the level of the papillary muscle. For each mouse, LV end-diastolic diameter (LVEDD), LV end-systolic diameter (LVESD), LV end-diastolic posterior wall thickness (LVEDPW) and intra-ventricular septum dimension at end-diastole (IVSED) were quantified, and LV mass ascertained in mg with the following equation^31^,^32^:


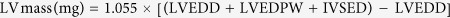


A visual illustration of methods of mouse cardioechography can be found in a video publication by Respress and Wehren^33^.

### VSMC isolation

Mouse VSMC were isolated from aortic and mesenteric arteries, including their secondary branches as described before^22^,^23^.

### siRNA transfection and reverse transcription-quantitative polymerase chain reaction (RT-qPCR)

The sequences of *Grip1, Pdg-rgs3, and Disheveled* siRNA and siRNA transfection procedures are described in our previous publication^22^. Their mRNA levels in VSMCs were measured by RT-qPCR; the primer sequences and qPCR cycling program are detailed before^22^. β-actin mRNA served as internal control. Samples were tested in triplicate, and the data were expressed as signal ratios of test gene mRNA/α-actin mRNA.

### VSMC contractility

VSMC contractility was measured as described before^22^,^23^. Briefly, the cells were cultured in wells coated with goat anti-mouse EFNB3 Ab (sc-7281, Santa Cruz Biotechnology, Dallas, Texas) or control goat IgG (2 μg/ml during coating). After 3–4 days, they were stimulated with PE (20 μmol/L), and photographed continuously for 15 min at a rate of 1 picture per min. Fifteen or more cells were randomly selected, and their length was measured at each time point with Zeiss Axiovision software. Percentage contraction was calculated as follows:


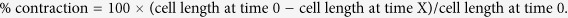


### Ethics Statement

This study was carried out in strict accordance with the recommendations in the Guide for the Care and Use of Laboratory Animals of the National Institutes of Health. The protocol was approved by the Institutional Committee on Animal Care of CRCHUM (Permit Number: 4I14033JWs). All surgery was performed under isoflurane anesthesia, and all efforts were made to minimize suffering. Informed consent was obtained from all the subjects used in this study.

## Results

### Association of SNPs in the EPHB/EFNB system with BP phenotypes

**Figure 1:**
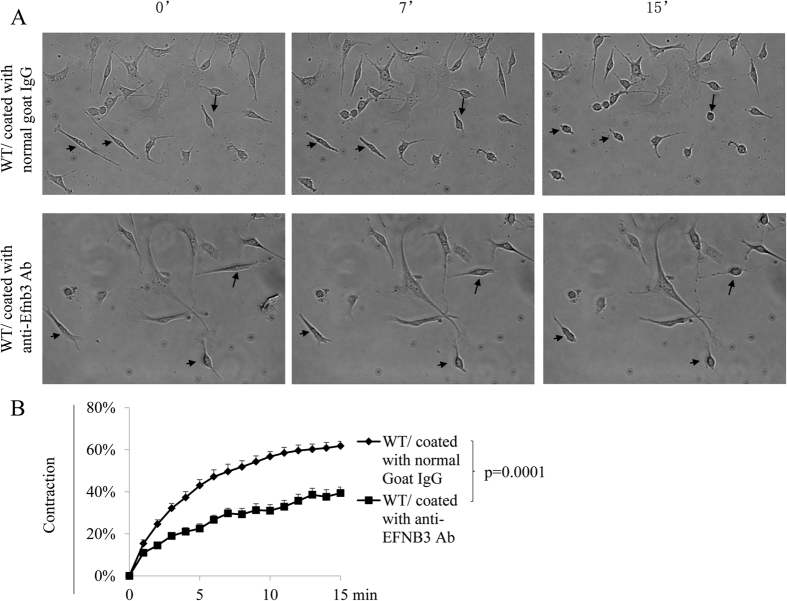
Crosslinking EFNB3 on VSMCs results in decreased contractility. VSMCs from female WT mice were cultured in wells coated with goat anti-mouse EFNB3 Ab or control goat IgG (2 μg/ml during coating) for 4 days. The cells were then stimulated with PE (20 μmol/L), and their percentage contraction was recorded by microscopy. (**A**) Micrographs showing the contraction of WT VSMCs in the presence or absence of solid phase anti-EFNB3. Upper row: WT VSMCs cultured in wells coated with control goat IgG (20 μg/ml for coating). Lower row: WT VSMCs cultured in wells coated with goat anti-mouse EFNB3 Ab. The images were taken at 0, 7 and 15 min after PE stimulation. Arrows mark the same cells in each row at different time points, to show the shortening of the cells. (**B**) Reduced contractility of WT VSMCs cultured in the presence of solid phase anti-EFNB3 Ab. VSMCs from female WT mice were cultured in wells coated with normal goat IgG or goat anti-mouse EFNB3 Ab for 4 days. The cells were then stimulated with PE, and were imaged at one frame per min for 15 min. Means ± SD of the percentage contraction during a 15-min period are reported. The percentage contraction is calculated as follows. % contraction = length of cells at a given timepoint/length of the cells at time 0. The data were analyzed with one-way ANOVA, and *p*-value is indicated. The experiment in this figure was repeated three times, and representative data are shown.

Our previous studies^22^,^23^ along with some of our unpublished observations indicate that molecules in the EPHB and EFNB families (*e.g.,* EPHB6, EFNB1, EFNB2, and EFNB3) and a certain adaptor protein (GRIP1) within their signalling pathways are novel factors that can modulate BP in mice. The relevance of these molecules in human BP regulation was investigated. The IBPC conducted a meta-analysis of genome-wide association scanning (GWAS) in 69,395 individuals of European ancestry in 29 cohorts from European and North American countries^25^. Two and a half million genotyped or imputed SNPs were tested for their association with SP and DP in these individuals. We queried the results of this meta-analysis for association of 528 SNPs in *EPHB6, EFNB2, EFNB3* and *GRIP1* genes with systolic or diastolic pressure in these individuals. *EFNB1* was not included in the analysis because it is an X-linked gene, and its SNP information is not available in the IBPC dataset. The *p*-values of these SNPs for their association with systolic or diastolic pressure are illustrated in Supplementary Figure 1 (S. Fig. 1). Table 1 summarizes the names of SNPs with the most significant association and their *p*-values. The minimum observed *p*-value for any association (0.000389) was for the association of SNP rs1495496 located within the *GRIP1* gene with diastolic pressure. This value approaches the critical *p*-value of 0.000302. For the other 3 genes analyzed*, i.e.,* EPHB6, EFNB2 and EFNB3, the minimum *p*-values of their SNPs did not approach the critical *p*-value. The implications of these findings are elaborated in the Discussion.

### Small artery resistance in EFNB3 KO mice *in vivo*

EFNB3 is an EPHB6 ligand, and its function might contribute to the BP phenotype observed in *Ephb6* KO mice^22^. We assessed several BP related parameters in *Efnb3* KO mice (called KO mice hereafter) using echocardiography.

BP is a function of CO, blood volume and blood vessel flow resistance. Echocardiography was employed to examine CO, the PI (a parameter reflecting blood vessel flow resistance) of carotid arteries, and LV mass in live KO and WT mice. Due to technical limitations, we were not able to conduct echocardiography in smaller arteries. CO in male (Table 2) and female (Table 2) KO mice was comparable to that in WT controls, indicating that this parameter, which could affect BP, is not under the influence of EFNB3. However, the left carotid PI in female KO (Table 2) but not male KO (Table 2) mice was significantly higher than that of WT counterparts (p = 0.0218), reflecting heightened resistance of these small arteries. LV mass increased significantly in female but not in male KO mice, compared to their WT counterparts (p = 0.0397, Table 2). This hypertrophy might be the result of augmented cardiac workload in female KO mice to overcome the heightened blood flow resistance of their small arteries.

### GRIP1 knockdown by siRNA cancelled the effect of solid phase anti-EFNB3 Ab on the contractility of WT VSMCs

**Figure 2:**
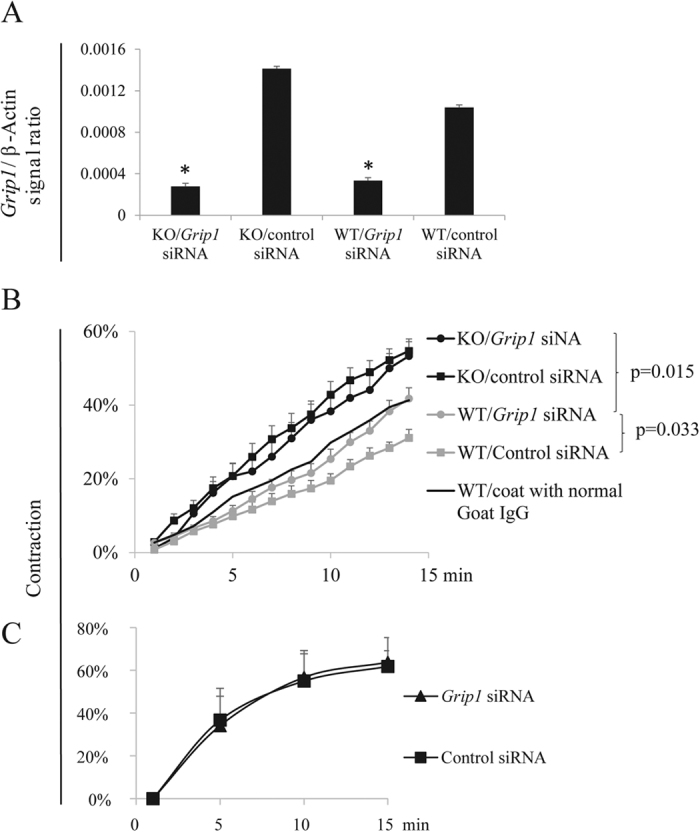
GRIP1 in the EFNB3 reverse signaling pathway in VSMCs. Experiments in this figure were repeated more than twice, and representative data are shown. (**A**) Effective mRNA knockdown of Grip1 by siRNA. VSMCs from female WT mice were transfected with siRNAs of a particular gene or control siRNA, as indicated. After 24-h culture, the cells were harvested and the mRNA expression of each gene was determined by RT-qPCR. The data are expressed as means ± SD of the ratios of the target gene signal versus the β-actin signal. The data were analyzed by one-way Student’s *t* test. *p < 0.05. (**B**) GRIP1 knockdown by siRNAs reverses the effect of solid-phase anti-EFNB3 Ab on reducing VSMC contractility. VSMCs from female WT and KO mice were cultured in wells coated with goat anti-mouse EFNB3 Ab (2 μg/ml during coating). After 2 days, they were transfected with siRNAs targeting *Grip1*, or with control siRNA. On day 4 of culture, they were stimulated with PE (20 μmol/L), and their percentage contraction was recorded. Means ± SD of the percentage are reported. The thin line represents mean contractility of VSMCs cultured in well without coating of anti-EFNB3 Ab as an additional control; for better viewing, SD was not added to the line. The data were analyzed with one-way ANOVA followed by ad *hoc* analysis, and p-values of significant difference are indicated. (**C**) Grip1 siRNA in the absence of EFNB3 reversing signaling had no effect on VSMC contractility. VSMCs from female WT mice were cultured in plain wells without Ab coating. They were transfected with Grip1 or control siRNA and then stimulated with PE as described in (**A**). Mean ± SD of percentage contraction are shown. The data were analyzed with one-way ANOVA but not statistically significant difference between the test and control groups is found.

The echocardiography results suggest that the default function of EFNB3 is vessel relaxation, and hence the EFNB3 KO carotid artery manifested increased resistance. To prove this at the cellular level, we triggered EFNB3 reverse signalling by incubating WT VSMCs in wells coated with anti-EFNB3 Ab and tested their contractility. Such solid phase anti-EFNB3 Ab serves as an agonist to EFNB3, because it acts at the bottom of the cells and will not block putative EFNB3/EPH interaction which might exist at the vertical side of neighboring VSMCs. Further, during to the low density used in culture, such fraternal cell interaction is rare. A similar strategy of solid phase anti-EFNB1 Ab as an agonist to trigger EFNB1 reverse signalling in VSMCs has been previously published^23^. As shown in Fig. 1A and B, WT VSMCs crosslinked with anti-EFNB3 Ab presented significantly reduced contractility, compared to those cultured in wells coated with normal goat IgG. On the other hand, KO VSMC contractility was not affected by anti-EFNB3 crosslinking, as expected, because they have no EFNB3 expression (data not shown). These results corroborate that of echocardiography and confirm that EFNB3 reverse signalling leads to reduced VSMC contractility. The solid phase anti-EFNB3 Ab indeed triggered signalling in VSMCs in that their ERK phosphorylation upon PE stimulation was increased (S. Fig. 2).

**Figure 3:**
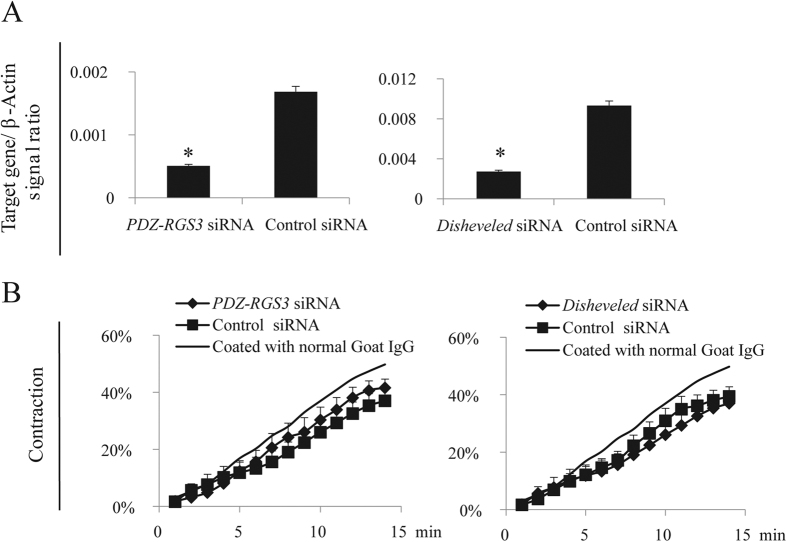
DISHEVELLED and PDZ-RGS3 are not in the EFNB3 reverse signaling pathway in VSMCs. Experiments in this figure were repeated more than twice, and representative data are shown. (**A**) Effective mRNA knockdown of *Disheveled* and *PDZ-RGS3* by siRNA. VSMCs from female WT mice were transfected with siRNAs of Disheveled and PDZ-RGS3 as described in Fig. 2. The mRNA expression of each gene was determined by RT-qPCR. The data are expressed as means ± SD of the ratios of the target gene signal versus the β-actin signal. The data were analyzed by Student’s *t* test. *p < 0.05. (**B**) Dishevelled and PDZ-RGS3 knockdown by siRNAs had no effect on WT VSMC contractility. Contractility of VSMCs from female WT mice were assessed in the presence of EFNB3 reverse signalling and DISHEVELLED/PDZ-RGS3 knockdown, as described in Fig. 2B. The data were analyzed with one-way ANOVA but no significant difference was observed.

EFNBs had no enzymatic activity. However, EFNBs engage adaptor proteins to link their intracellular tails to various signalling pathways. *Grip1* siRNA was used to knock down the expression of this adaptor protein in the WT and KO VSMCs, as it is known to associate with EFNBs^34^,^35^,^36^. The effectiveness of mRNA knockdown was verified by RT-qPCR (Fig. 2A). EFNB3 expression in the WT VSMCs was not affected by the knockdown (S. Fig. 3). WT VSMCs were cultured in wells coated with anti-EFNB3 Ab to invoke EFNB3 reverse signalling, and KO VSMCs were used as controls. In WT VSMCs, such treatment (Fig. 2B) dampened VSMC contractility of WT VSMCs transfected with control siRNA, but such dampening effect was reversed by *Grip1* siRNA (Fig. 2B). KO VSMCs cultured in wells coated with anti-EFNB3 Abs had a higher contraction, as they had no EFNB3 and were not affected by anti-EFNB3-evoked reverse signalling, which could reduce contractility. As expected, *Grip1* siRNA knockdown did not affect KO VSMC contractility, because GRIP1 was not utilized by the KO VSMCs. The effect of GRIP1 knockdown on the augmentation of WT VSMC contractility depended on the existence of EFNB3 reverse signalling, as *Grip1* siRNA had no effect on the contractility of WT VSMCs cultured in wells without anti-EFNB3 Ab coating (Fig. 2C). DISHEVELLED and PDZ-RGS3 are two other proteins capable of associating with the EFNB3 intracellular tail^35^,^36^. However, their knockdown by siRNA had no effect on WT VSMC contractility (Fig. 3), suggesting that they were not involved in EFNB3 reverse signalling in VSMCs.

These results corroborate the finding of the human genetic study, indicating that GRIP1 is involved in EFNB3 reverse signalling and VSMC contractility regulation, and it is hence a factor modulating blood pressure.

## Discussion

EPHs and EFNs perform critical functions in many tissues and organs. In our previous studies, we found that *Ephb6* KO and *Efnb1* KO mice have augmented BP^22^,^23^. In the current investigation, we provided additional human genetic evidence showing that the mutations in GRIP1, a molecule in the EPHB6/EFNB signalling pathway and an adaptor protein capable of binding to EFNBs, were associated with human blood pressure phenotype. We also provided mouse experimental data showing that the default function of EFNB3, one of the ligands of EPHB6, was to reduce arterial tone, and such function depended on signalling via GRIP1, corroborating the human genetic findings.

As of today, no genes in the EPHB/EFNB family have been identified as hypertension risk genes in several large-scale GWAS^25^,^37^,^38^,^39^,^40^,^41^,^42^,^43^,^44^. There are a couple of possible reasons. 1) The contribution of genes in the EPHB/EFNB family to the BP phenotype might be relatively small, and the possible association is rendered undetectable due to heavy statistical penalties of multiple testing in GWAS. 2) In order to reduce the effect of type 2 error resulting from multiple-testing correction in genome-wide association studies, the IBPC study has assembled very large samples by combining cohorts from many sub-studies. The hypothesis is that increasing sample size will increase power, allowing the decrease in both type 1 and type 2 errors. However, *p*-value is controlled by both sample size and effect size (odds ratio) and, of the two, a slight decrease in effect size has a more dramatic impact on *p*-value. Assembly of large disparate cohorts does increase sample size but it also introduces increased heterogeneity, both phenotypic and genetic. Such heterogeneity may reduce the average effect size.

We conducted a more focused query of the IBPC dataset, using only 4 genes in the EPHB/EFNB signalling pathway, and discovered that a SNP, rs1495496, located between exons 22 and 23 of *GRIP1*, had a *p*-value of 0.000389 for its association with DP, which approaches the Bonferroni corrected critical *p*-value 0.000302. This finding is very promising since Bonferroni correction by itself was deemed to be quite conservative. Considering our observation that EPHBs/EFNBs influence vessel tone in mice, the possible association of this SNP in *GRIP1* gene with DP seems logical. Why does only a SNP from *GRIP1* approach significance and not those from 3 *EPHB6/EFNBs* queried? GRIP1 is situated in a node of EPHB6, EFNB1 and EFNB3 reverse signalling pathway; it thus probably carries more weight than each individual EPHB or EFNB member and, hence, is easier to detect for its associations with BP phenotypes. Our recent published results^22^,^45^ and unpublished observations in animal models indicate that the effect of EPHBs and EFNBs on blood pressure regulation is not only sex-dependent, but also sex hormone level-dependent. It is possible that in a cohort stratified by sex and sex hormone levels, more significant association of BP phenotypes with SNPs from *GRIP1* and *EPHB/EFNB* family members will be detected in humans, and this will fully establish the relevance of our findings from animal models to human blood regulation.

Our cardioechogrphy data using EFNB3 KO mice suggest a default sex/sex hormone-dependent vasolaxative role of EFNB3 in that its deletion led to increased blood flow resistance in the carotid artery of female KO but not male KO mice, compared to their WT counterparts. Such increased resistance is not due to developmental changes of the arteries in the KO mice, as we found no abnormalities in the aorta and mesenteric arteries in terms of histology and diameters (S. Fig. 4).

Using telemetry, we have recently revealed that female but not male EFNB3 KO mice have increased BP, but after ovariectomy, the female KO BP returns to the normal range^46^. This finding corroborates the data from cardioechography, suggesting that after EFNB3 deletion, increased vascular tone results in increased BP, but such effect is sex- and sex hormone level-dependent.

We demonstrated EFNB3 can modulate VSMC contractility by reverse signalling. EFNB3 is a transmembrane protein without enzymatic activity in its intracellular tail. How does it regulate VSMC contractility? We have demonstrated that the association of EFNB3 with GRIP1 is critical for its function in regulating VSMC contractility. In VSMCs, EFNB3 might also associate with other so-far unidentified binding proteins. During reverse signaling, some of these proteins might interact with and modulate the functions of other pathways that control VSMC contractility, the MLCK pathway being one of them. The intermediate signalling molecules between EFNB3/GRIP1 and MLCK/MLC remain to be identified.

In our previous publication, we reported that deletion of EPHB4 in mice results in reduced BP, and GRIP1 is involved in the signaling between EPHB4 and its EFN ligands^45^, most likely its major ligand EFNB2. In this study, we showed that GRIP1 is also involved in EFNB3 signaling, the deletion of which leads to increased BP. How do we explain that the same molecule is mediating signals that resulting opposite phenotype in the same type of VSMCs? In the biological system, it is not uncommon that a molecule can mediate multiple different functions, depending to the status of a cell and the different upstream signals it receives. For example, β-catenin can mediate many different cellular events, such as cell adhesion, stem cell renewal, asymmetric cell division, etc., depending which binding partners it associates with^46^. GRIP1 is no different in this sense: it can mediate signaling leading to increased or decreased VSMC contraction, depending on its upstream signaling: from EFNB (the preferred ligand of EPHB4) or EFNB3, respectively.

What is the possible mechanism by which estrogen in concert with EFNB3 modulates VSMC contractility? We have demonstrated that estrogen augments KO but not WT VSMC contractility, and this is mediated by its nongenomic effect via cell surface receptor GPR30. GRP30 can directly regulate ERK activity^47^, which in turn controls VSMCs contractility^48^. As EFNB3 reverse signalling can activate ERK as shown in S. Fig. 2, ERK or molecules in this category which modulates VSMC activity both in the presence or absence of EFNB3, can function as a node on which EFNB3 and GPR30 signalling pathways converge. This could be a possible mechanism by which EFNB3 and estrogen act in concert in regulating VSMC contractility. Our additional study revealed that EFNB3 deletion upregulates GPR30 expression and the upregulation is at the transcription level^49^. There was no evidence of direct interaction between EFNB3 and GPR30, according to fluorescent resonance energy transfer assays (data not shown).

Our research on the roles of EPHBs/EFNBs in VSMC contractility has revealed a group of previously unknown molecules capable of regulating vascular tone and blood pressure. Among this group of molecules, we have shown that the default function of EPHB6, EFNB1 and EFNB3 is to reduce vascular tone^22^,^23^,^49^, and thus their deletion leads to increased vessel contractility and blood pressure. Our most recent finding indicates that among the EPHBs and EFNBs, there are another group of molecules such as EPHB4 and its major ligand EFNB2, whose default function is opposite*, i.e*., to enhance the vascular tone^45^,^50^; consequently, their deletion lead to reduced blood pressure. These opposing forces of different member of EPHBs and EFNBs are like Yin and Yang and probably play a role for the fine tuning of the vascular tone. A better understanding of the physiological relevance and mechanisms of action of these molecules for their role in vascular contractility and blood pressure regulation will afford us a novel personalized therapeutic approach to blood pressure management. For example, female hypertensive patients with EFNB3 mutations could be identified by genetic testing, and for this subpopulation of patients, avoidance of oral contraceptive/hormone replacement could reduce their hypertension risks.

## Additional Information

**How to cite this article**: Wang, Y. *et al*. The role of GRIP1 and ephrin B3 in blood pressure control and vascular smooth muscle cell contractility. *Sci. Rep.* **6**, 38976; doi: 10.1038/srep38976 (2016).

**Publisher's note:** Springer Nature remains neutral with regard to jurisdictional claims in published maps and institutional affiliations.

## Supplementary Material

Supplementary Data

## Figures and Tables

**Table 1:** Association of SNPs in the EPHB6/EFNB system with BP phenotypes in 69,396 human subjects.

Locations of 4 genes for which 528 SNPs were tested						
Gene	Build 36			Build 37		
	Location		Size (kb)	Location		size (kb)
*EPHB6*	chr7: 142,252,914–142,288,969		36.06	chr 7: 142,542,792–142,578,847		36.06
*EFNB2*	chr13: 105,930,097–105,995,338		65.24	chr 13: 107,132,079–107,197,388		65.31
*EFNB3*	chr17: 7,539,245–7,565,418		26.17	chr 17: 7,598,520–7,624,693		26.17
*GRIP1*	chr12: 65,019,066–65,369,020		349.95	chr 12: 66,731,211–67,082,925		351.71
**Minimum p-values from IBPC meta-analysis among SNPs examined within EPHB6, EFNB2, EFNB3 and GRIP1 genes**						
**Gene**	**Number of SNPs examined**	**LD Blocks r2 = 0.8**	**Diastolic Pressure**		**Systolic Pressure**	
			**SNP**	**p-value**	**SNP**	**p-value**
*EPHB6*	48	4	rs1009848	0.0373	rs2299557	0.404
*EFNB2*	54	24	rs2057408	0.077	rs9520087	0.218
*EFNB3*	6	6	rs3744258	0.201	rs3744258	0.191
*GRIP1*	420	132	rs1495496	0.000389	rs1495496	0.00144
Total	528	166	Critical p value: 0.05/166 = 0.0003012			

**Table 2:** Echocardiographic analysis of CO, carotid artery resistance and left ventricle mass of Efnb3 KO and WT mice.

Mouse Type	Age	Cardiac output (ml/min)	Left Carotid PI	Right Carotid PI	LV Mass (mg)
**Echocardiographic parameters of male Efnb3 KO mice**					
KO male	15wk	14.65	0.75	0.72	103.7
KO male	15wk	21.46	0.87	0.68	125.8
KO male	15wk	24.79	0.73	0.78	154.1
KO male	15wk	17.23	0.74	0.70	91.7
KO male	15wk	14.93	0.78	0.78	89.7
KO male	14wk	18.2	0.73	0.72	133.6
**KO mean**		**18.54**	**0.77**	**0.73**	**116.4**
**SD**		3.939	0.0533	0.041	25.65
WT male	14wk	21.5	0.83	0.84	116.0
WT male	15wk	15.33	0.74	0.70	140.5
WT male	15wk	23.52	0.75	0.77	115.6
WT male	15wk	18.21	0.73	0.72	133.6
WT male	14wk	31.85	0.77	0.86	115.0
WT male	14wk	28.57	0.77	0.81	145.1
WT male	14wk	24.19	0.74	0.76	152.7
WT male	14wk	30.95	0.79	0.83	175.6
**WT mean**		**24.265**	**0.77**	**0.78**	**126.4**
**SD**		5.92	0.030	0.047	12.57
**p value (t test)**		0.0637	0.9075	0.08200	0.4967
**Echocardiographic parameters of female Efnb3 KO mice**					
KO female	15wk	21.89	0.75	0.78	112.3
KO female	15wk	16.29	0.77	0.8	92.5
KO female	14wk	20.16	0.81	0.81	121.1
KO female	15wk	27.57	0.76	0.77	143.3
KO female	15wk	20.54	0.94	0.76	130.5
KO female	14wk	27.71	0.75	0.73	134.3
KO female	15wk	19.13	0.8	0.77	135.8
KO female	14wk	18.38	0.81	0.82	122
**KO mean**		**21.45**	**0.79**	**0.78**	**123.9**
**SD**		4.153	0.062	0.029	16.04
WT female	14wk	33.49	0.72	0.72	109
WT female	14wk	18.92	0.69	0.67	118.1
WT female	14wk	29.03	0.71	0.8	110.9
WT female	14wk	28.06	0.81	0.79	95.9
WT female	14wk	15.96	0.68	0.76	114.6
WT female	14wk	22.46	0.7	0.74	72.2
**WT mean**		**24.65**	**0.71**	**0.74**	**93.4**
**SD**		6.664	0.047	0.048	29.98
**p value (t test)**		0.289	**0.0218**	0.132	**0.0397**

BP-related echocardiographic parameters of individual *Efnb3* KO and WT mice are reported. Means ± SD are shown at the end of each group, and *p* values (unpaired one-way Student’s *t* test) are indicated at the bottom of Tables 2. PI: Pourcelot index; LV: left ventricle.
