# Supplementary material for: The role of GRIP1 and ephrin B3 in blood pressure control and vascular smooth muscle cell contractility
Source: Sci Rep. 2016 Dec 12;6:38976. doi: 10.1038/srep38976 (PMC5150233; doi:10.1038/srep38976)
Supplement: Supplementary Data [file srep38976-s1.doc]

# The role of GRIP1 and ephrin B3 in blood pressure control and vascular smooth muscle cell contractility

^1^Yujia Wang, ^1^Zenghui Wu, ^1^Hongyu Luo, ^1^Junzheng Peng, ^1^John Raelson, ^2^Georg B. Ehret, ^3^Patricia B. Munroe, ^1^Ekatherina Stoyanova, ^1^Zhao Qin, ^1^Guy Cloutier, ^1^W. Edward Bradley, ^4^Tao Wu, ^4^Jian-Zhong Shen, ^4^Shenjiang Hu and ^1,5^Jiangping Wu

From the ^1^Research Centre and ^5^Nephrology Service, Centre hospitalier de l’Université de Montréal (CHUM), Montreal, Quebec H2X 0A9, Canada; ^2^Center for Complex Disease Genomics, McKusick-Nathans Institute of Genetic Medicine, Johns Hopkins University School of Medicine, Baltimore, Maryland 21205, USA; ^3^Clinical Pharmacology and The Genome Centre, William Harvey Research Institute, Barts and The London School of Medicine and Dentistry, Queen Mary University of London, London EC1M 6BQ, UK; ^4^Institute of Cardiology, First Affiliated Hospital, Zhejiang University Medical College, Hangzhou, 310003, China

# Short title: The role of GRIP1 and EFNB3 in blood pressure regulation

Address correspondence to: Dr. Jiangping Wu, CHUM Research Center (CRCHUM), 900 Saint-Denis Street, Rm. R12.428, Montreal, Quebec H2X 0A9, Canada. Telephone: (514) 890-8000 Extension 25164, Fax: (514) 412-7944, e-mail: [jianping.wu@umontreal.ca](mailto:jianping.wu@umontreal.ca)


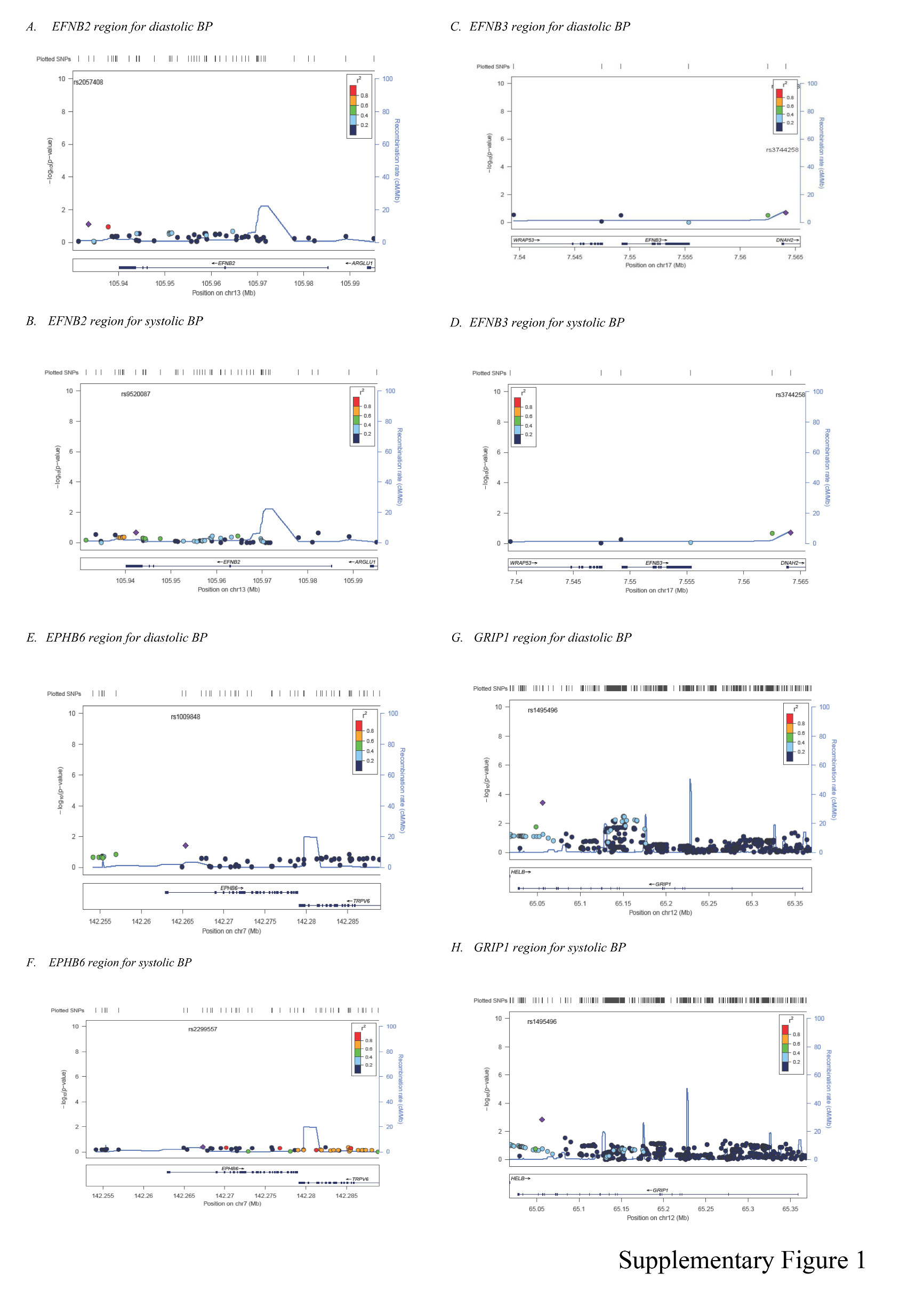
**SUPPORTING INFORMATION**

*Supplementary Figure 1. LocusZoom plots of -log10 p-values (left hand vertical axis) from IBPC meta-analysis of specific queried genes*

The positions of all SNPs in the query are indicated at the top of the plot as short, thin vertical lines, and as diamonds and circles in the plots. Diamonds represent reference SNPs with the highest -log10 p-value in the region, and their names are indicated above the diamonds. R2 refers to LD between the reference SNP and respective SNPs within the region. Blue lines refer to recombination rates (right hand vertical axis) in centimorgans/megabase at each position. Thus, peaks in blue lines indicate low LD regions of high recombination rates or recombination hotspots.


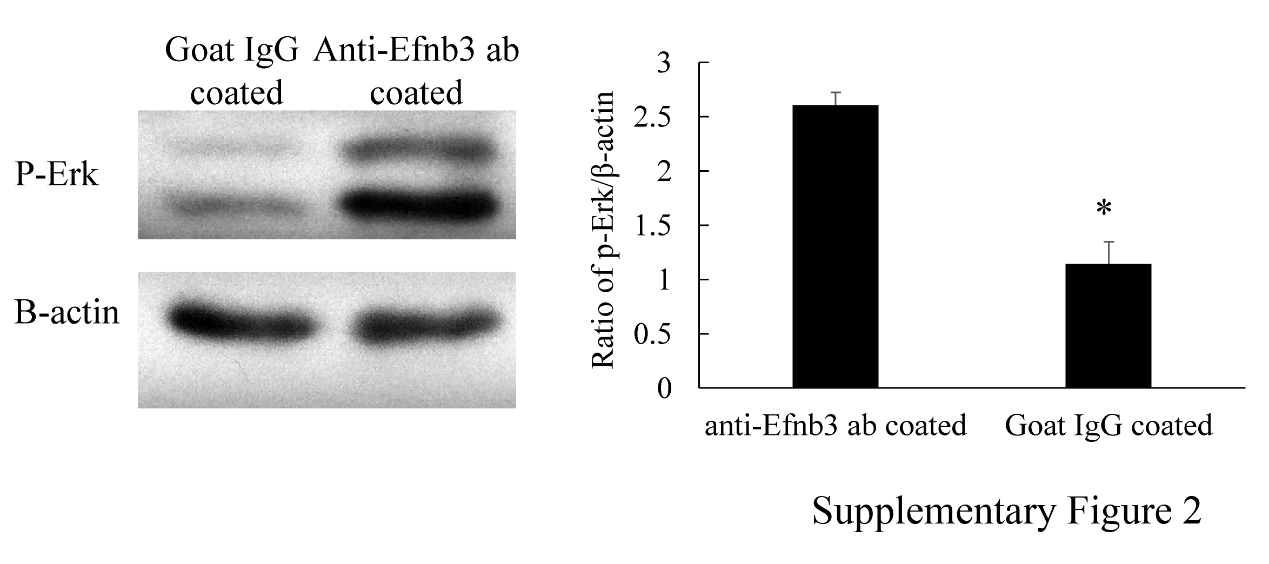


*Supplementary Figure 2. Reduced ERK phosphorylation in WT VSMCs stimulated with solid phase anti-EFNB3 Ab*

VSMCs from female WT mice were cultured in wells coated with anti-EFNB3 Ab, and then stimulated with PE, as described in Figure 1. The cells were harvested 3 sec after PE stimulation, and their ERK phosphorylation was determined by Western blotting. The experiment was conducted 3 times and a representative result is shown.


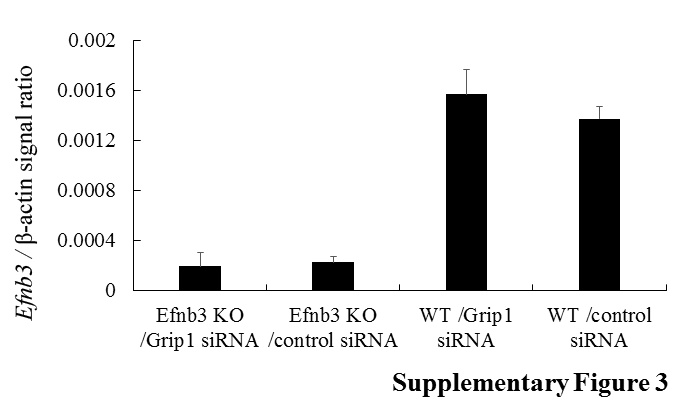


*Supplementary Figure 3. Unaltered EFNB3 mRNA expression in WT VSMCs transfected with Grip1 siRNA*

VSMCs from female WT mice were transfected with control and EFNB3 siRNA. After 48 h, their EFNB3 mRNA expression was determined by RT-qPCR. The mean + SD of the ratios between EFNB3 and β-actin signals in two independent experiments are presented. No significant difference is observed between control and EFNB3 siRNA transfected samples (Student's *t*-test).


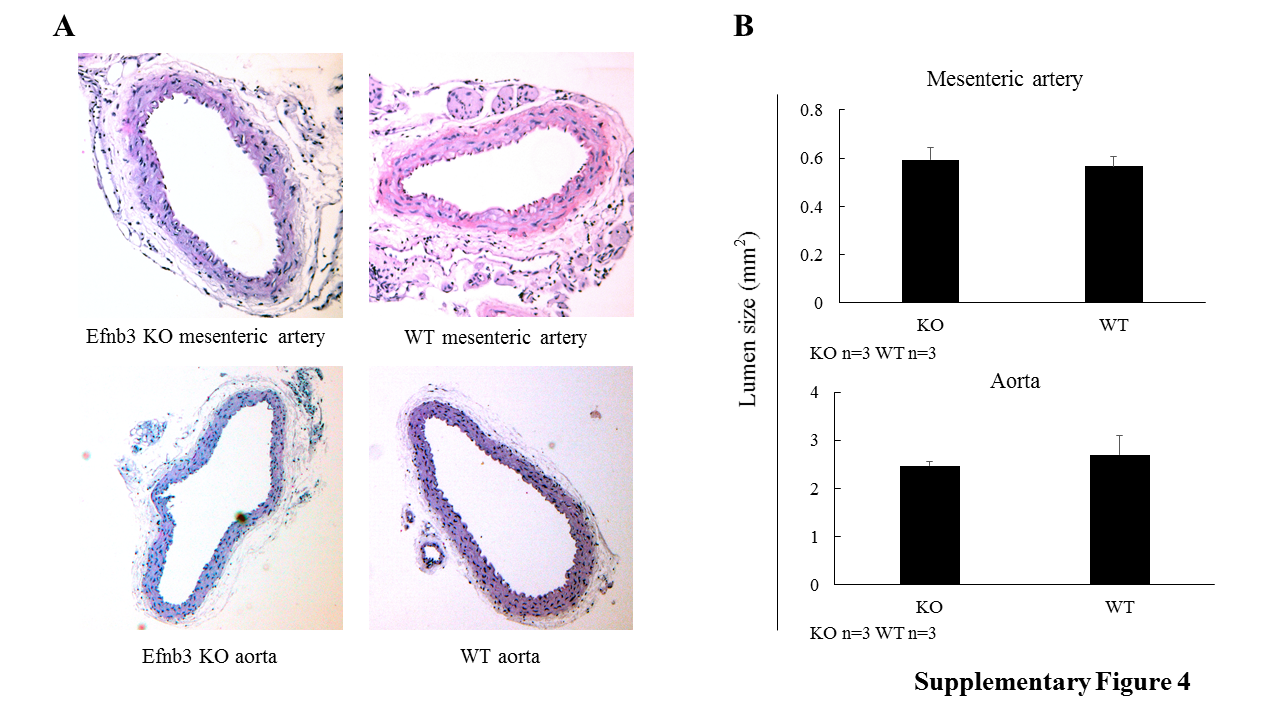


*Supplementary Figure 4. Histology and lumen diameters of KO and WT vessels*

*A. HE-stained sections of the aorta, and mesenteric of WT and KO mice*

*B. Lumen sizes of WT and KO vessels*

The lumen sizes of the aorta, and mesenteric artery of female WT and KO mice were measured using Image J and mean + SD of data from 3 WT and 3 KO mice of similar age are presented. No significant difference is found (Student's *t* test).
